# Consumption of coffee and tea and the risk of developing neurodegenerative diseases: a cohort study in the UK biobank

**DOI:** 10.1186/s12937-026-01291-0

**Published:** 2026-03-03

**Authors:** Yixiang Lin, Yisen Shi, Lina Chen, Zhuoli Chen, Huilin Zhong, Yuxuan Zhong, Ruitian Zeng, Xinyan Chen, Fabin Lin, Guoen Cai

**Affiliations:** 1 Institute of Clinical Neurology, Institute or Neuroscience,Department of Neurology, Center for Cognitive Neurology, Fujian Medical University Union Hospital, 29 Xinquan Road, Fuzhou, 350001 China; 2 Fujian Institute of Geriatrics, Fujian Medical University Union Hospital, 29 Xinquan Road, Fuzhou, 350001 China; 3 Fujian Key Laboratory of Molecular Neurology, Fujian Medical University, 88 Jiaotong Road, Fuzhou, 350001 China; 4 Department of Neurosurgery, Fujian Medical University Union Hospital, 29 Xinquan Road, Fuzhou, 350001 China

**Keywords:** Coffee, Neurodegenerative disease, Tea

## Abstract

**Background:**

Coffee and tea are among the most consumed drinks worldwide. Increasing evidence indicates an association between coffee or tea intake and neurodegenerative diseases. However, most studies have focused on the association of coffee or tea alone; studies on the interactive associations between coffee and tea and neurodegenerative diseases are few. Therefore, this study aimed to explore the individual or interactive associations between coffee and tea intake and neurodegenerative diseases and their various subtypes.

**Methods:**

This study included 134,425 participants without neurodegenerative diseases at baseline in UK Biobank. A total of 6483 participants developed neurodegenerative diseases during a median follow-up time of 13.5 years. The median daily coffee intake was two cups, and the median daily tea intake was three cups. A restricted cubic spline was used to explore the nonlinear associations between coffee or tea intake and neurodegenerative diseases. The individual or interactive associations between coffee and tea intake and neurodegenerative diseases were assessed using the Cox proportional-hazards model.

**Results:**

An individual association was noted between coffee or tea intake and neurodegenerative diseases. A significant J-shaped association was found between coffee intake and all-cause neurodegenerative diseases (Pnonlinear = 0.004) and vascular neurodegenerative diseases (Pnonlinear = 0.023), with increased risk at higher consumption. Moreover, a nonlinear association was observed between tea intake and all-cause neurodegenerative diseases (Pnonlinear = 0.004), vascular neurodegenerative diseases (Pnonlinear = 0.031), other neurodegenerative diseases (Pnonlinear = 0.002), and vascular dementia (VD) (Pnonlinear = 0.026). Furthermore, a significant interactive association was noted between coffee and tea intake among all-cause neurodegenerative diseases (Pinteraction = 0.004); Further, this interaction was also observed in Alzheimer’s disease (AD) (Pinteraction = 0.006).

**Conclusions:**

Excessive coffee consumption was significantly associated with an increased risk of all-cause neurodegenerative diseases and vascular neurodegenerative diseases. The results also showed that tea intake was associated with a reduced risk of all-cause neurodegenerative disease, vascular neurodegenerative disease, other neurodegenerative diseases, and VD. Moreover, coffee and tea had an interactive relationship with all-cause neurodegenerative diseases and AD, with specific combinations significantly associated with reduced risk of disease onset.

**Supplementary Information:**

The online version contains supplementary material available at 10.1186/s12937-026-01291-0.

## Introduction

Neurodegenerative diseases are a set of neurological disorders resulting from a gradual loss of neurons in the central or peripheral nervous system. These neurons cannot effectively self-renew because of the nonregenerative nature of the terminal differentiation of neurons, leading to the breakdown of core communication circuits and, ultimately, impaired memory, cognitive, behavioral, sensory, or motor function [1]. The incidence of neurodegenerative diseases in most countries continues to gradually increase with the increase in the average age of the population [2]. There was an estimated 6.7 million Americans aged 65 years and older have Alzheimer’s disease (AD) in 2023. Without medical breakthroughs to prevent, slow, or cure AD, the number of people with AD will increase to 13.8 million by 2060. The total expenditures for people with dementia aged 65 years and older, including the provision of healthcare, long-term care, and end-of-life care services, reached $345 billion [3].

Given the scarcity of highly successful treatments for neurodegenerative diseases [4], identifying adjustable environmental risk variables and establishing preventive strategies are major public health challenges. Lifestyle habits have been shown to influence the risk of developing neurodegenerative diseases [5].

The association between coffee intake and neurodegenerative diseases is still not well understood. Coffee intake has been shown to reduce the risk of neurodegenerative diseases; the downstream metabolites of caffeine in coffee, such as theobromine, may modulate the effects of coffee on neurodegenerative diseases [6]. Also, a meta-analysis suggests that consuming one to four cups of coffee per day reduces the risk of dementia, but excessive daily coffee intake increases the risk [7]. A longitudinal study using logistic regression from the UK Biobank also concluded that people who drank more than six cups of coffee each day were more likely to develop dementia compared with those who did not drink coffee or drank in small amounts [8]. One study using the restricted cubic spline (RCS) suggests a nonlinear association between coffee intake and dementia [9].

Tea intake may also be associated with a reduced risk of certain neurodegenerative diseases such as dementia [10, 11]. A cross-sectional study, including adults aged 65 years or older in Zhejiang Province, eastern China, reported using logistic regression analysis that tea intake was related to a reduced risk of developing dementia, AD, and vascular dementia (VD) [11].

However, most current studies on the association between tea or coffee and neurodegenerative diseases focus on a single neurodegenerative disease. The association between both beverages and all-cause neurodegenerative diseases and specific subtypes have not been investigated. Additionally, fewer studies have explored their interactive relationship [9].

Therefore, this study was conducted to evaluate the link between coffee or tea intake and all-cause neurodegenerative diseases and their various subtypes [vascular neurodegenerative diseases, primary neurodegenerative diseases, other neurodegenerative diseases, Parkinson’s disease (PD), AD, VD, and amyotrophic lateral sclerosis (ALS)], relying on data from a large cohort at the UK Biobank. This study investigated linear and nonlinear associations of coffee or tea intake with neurodegenerative diseases using the Cox proportional-hazards model and RCS. The study also examined the interactive relationships between coffee and tea and neurodegenerative diseases.

## Materials and methods

### Study design and participants

The data used in this study were obtained from the UK Biobank, which is the world’s largest database of human biospecimens. Data on disease, lifestyle, and genotype were collected from approximately 50 million participants in the UK aged 40–69 years between 2006 and 2010 [12]. In 22 study centers across England, Wales, and Scotland, participants provided the aforementioned information through touchscreen questionnaires, interviews, physical and functional measurements, and genomic and biological evaluations. The study was approved by the North West Multi-center Research Ethics Committee, the National Information Management Committee, and the UK Government. Informed consent was obtained from all UK Biobank participants.

**Fig. 1:**
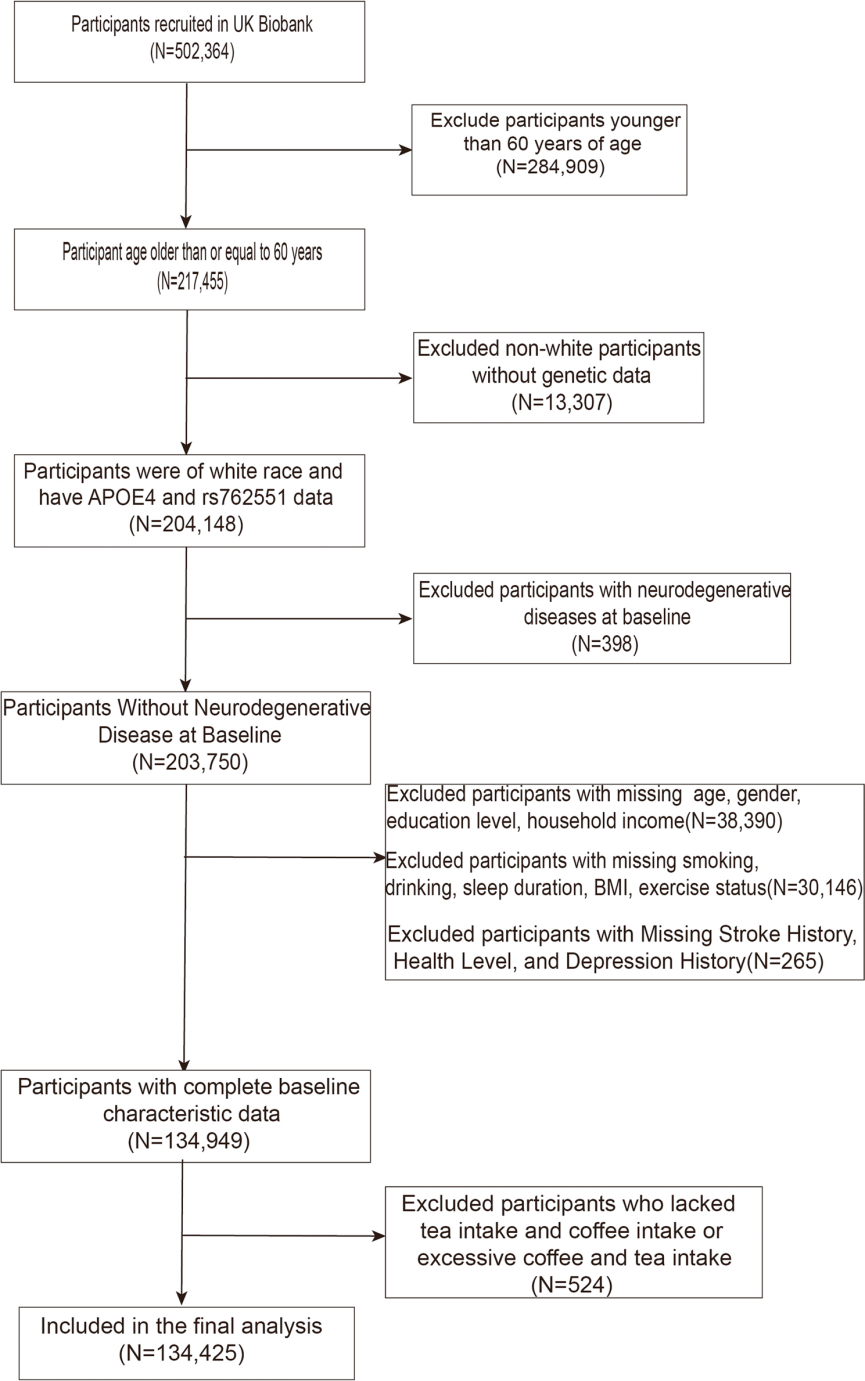
Selection of study participants in the UK Biobank

This study included 502,364 participants in the UK Biobank. As neurodegenerative diseases are prevalent in older people, we selected participants in the UK Biobank older than 60 years for this study. Previous studies showed that CYP1A2 gene activity varied across populations, and participants in the UK Biobank were primarily white. Hence, we chose white people for the study [13, 14]. In addition, we excluded participants who consumed more than 10 cups of coffee or 20 cups of tea per day to prevent the impact of their small numbers on the results of the experiment. After the final exclusion of participants with missing covariates, the study ultimately comprised 134,425 participants (Fig. 1).

### Assessment of coffee and tea intake

The participants were asked to complete the details on their daily coffee and tea consumption using a touchscreen at the UK Biobank’s experimental center [15, 16]. If the daily coffee and tea intake was less than one cup, it was defined as 0.5 cups in this study. The median daily coffee intake was two cups, and the median daily tea intake was three cups. In the coffee subgroup, the Cox proportional-hazards regression model was initially used to fit the hazard ratios (HRs) of all-cause neurodegenerative diseases for the population with different counts of coffee intake compared with the population with no coffee intake. Subsequently, HR curves for each coffee consumption count compared with the population with no coffee intake, were fitted using a locally weighted scatterplot smoothing with a bandwidth of 2/3 [17]. Structural breakpoints were then identified using the Chow test and piecewise linear regression using the “strucchange” and “segmented” R software packages. The breakpoints were considered as statistical thresholds. Tea intake was grouped in the same way as previously described for coffee intake. Final coffee intake was categorized into three groups: never, 0.5–5, and > 5 cups per day. Final tea intake was categorized into three groups: never, 0.5–3, and > 3 cups per day (Fig. S1).

### Determination of neurodegenerative diseases

The criteria for determining neurodegenerative diseases in this study were acquired from the International Classification of Diseases (ICD) 9th and 10th editions (ICD-9 and ICD-10). The disease data of participants were gathered from the UK Biobank initial evaluation and patient admission data, including self-reported medical conditions, procedures, and prescription data), besides death registrations from hospital-related data. We classified neurodegenerative diseases into all-cause neurodegenerative disease, vascular neurodegenerative diseases, primary neurodegenerative diseases, other types of neurodegenerative diseases, PD, AD, VD, and ALS [18, 19] (Table S1).

The primary outcome of this study was all-cause neurodegenerative disease, with secondary outcomes including vascular neurodegenerative disease, primary neurodegenerative disease, other neurodegenerative diseases, PD, AD, VD, and ALS.

### Assessment of covariates

The participants were required to fill in a questionnaire via a touchscreen. The questionnaire included age, sex, smoking status (no, former, and current), number of cigarettes smoked per day, alcohol drinking status (never, previous, and current), level of education (higher, lower secondary, upper secondary, and other), stroke history (yes or no), depression history (yes or no), hypertension history (yes or no), coronary heart disease history (yes or no), diabetes history (yes or no), cancer history (yes or no), sugar addition (yes or no), milk addition(yes or no), household income (less than £18,000, £18,000–£30,999, £31,000–£51,999, £52,000–1£00,000, and greater than £100,000), physical activity (if a person achieved the UK Physical Activity Guidelines for 150 min of walking or moderate physical activity, or 75 min of vigorous exercise each week), and sleep duration (< 7 h, 7–8 h, and > 8 h). A professional UK Biobank staff member measured the height and weight of the participants at the initial follow-up visit. Then, the body mass index (BMI) was obtained by dividing the weight (kg) by the square of the height (m). In this study, BMI was categorized into five groups: BMI < 18.5, BMI 18.5 to < 25, BMI 25 to < 30, BMI 30 to < 35, and BMI ≥ 35. Participants were asked to rate their overall health by answering, “In general, how would you rate your overall health?”. The overall health status was divided into four groups: excellent, good, fair, and poor. CYP1A2 enzyme participates in the metabolism of various drugs and is also the primary metabolic pathway for caffeine in humans. Moreover, different CYP1A2 genotypes may lead to differences in the rate of caffeine metabolism in the body [20]. The *CYP1A2* gene was used in the present study to classify the participants into two groups: the AA group, which was more efficient in metabolizing caffeine, and the AC and CC groups, which were less efficient [21]. APOE e4 genotypes were determined on the basis of rs7412 and rs429358, and the number of APOE e4 alleles per individual was categorized as none (e2/e2, e2/e3, or e3/e3), 1 (e3/e4 and e2/e4), and 2 (e4/e4) [22]. The healthy diet score was determined based on the participant’s weekly consumption of vegetables, fruits, fish, meat, and so on, on a scale from 0 to 5, where higher scores indicated a healthier diet [23]. Phenotypic age was determined using actual age and nine biomarkers associated with aging, including albumin, creatinine, and glucose. Phenotypic age acceleration was calculated by analyzing the residuals from the regression of phenotypic age on chronological age [24]. The higher the indicator, the quicker the aging process.

### Data analysis

We used the χ2 test for categorical variables and the analysis of variance (ANOVA) for continuous variables to evaluate the differences in baseline data across the coffee and tea categories. The Kruskal–Wallis test was used for non-normally distributed continuous variables. Follow-up time was calculated from the date participants attended the assessment center to the date of diagnosis of neurodegenerative diseases, death, loss to follow-up, or the last recorded admission date, whichever occurred first. The last recorded admission dates were October 31, 2022, for England, August 31, 2022, for Scotland, and May 31, 2022, for Wales.

First, we tested the nonlinear association between coffee and tea intake and neurodegenerative diseases using RCS; the knots of RCS were 5. We then transformed daily coffee and tea intake into categorical variables and explored the association between neurodegenerative diseases and coffee or tea intake using the Cox proportional-hazards model. In addition, we explored the interactive relationships between coffee and tea and neurodegenerative diseases. The daily coffee intake was combined with tea intake as a new variable to explore their interactive relationship on neurodegenerative diseases. The *P*-value used for heterogeneity corresponded to the chi-squared test statistic for the likelihood ratio test comparing models with and without interaction between coffee and tea. The results were presented as HR and 95% confidence interval (CI). Referring to previous studies [25], the covariates adjusted in the Cox proportional-hazards model were age, sex, smoking status, drinking status, sleep duration, stroke history, depression history, level of education, BMI, blood pressure status, overall health status, household income, physical activity status, and *CYP1A2* genotype. Tea consumption was also included as a covariate in the Cox proportional-hazards model when analyzing the association of coffee consumption with neurodegenerative diseases. Further, coffee consumption was included as a covariate in the Cox proportional-hazards model when analyzing the association between tea consumption and neurodegenerative diseases. A previous study showed that the *APOE* gene was a key risk factor for late-onset AD, which occurred after the age of 65 years [26]. Therefore, when analyzing the link between coffee or tea intake and AD, the *APOE* genotype of the participants was included in the model as a covariate.

In addition, we performed the following sensitivity analyses: [1] to exclude patients with neurodegenerative diseases of less than 2 years of duration and then analyze the association between neurodegenerative diseases and coffee or tea intake using the Cox proportional-hazards model; [2] to reassess the nonlinear association between coffee or tea intake and neurodegenerative diseases using RCS with the four knots; and [3] to investigate whether the CYP1A2 gene modulated the association between coffee and neurodegenerative diseases. The subgroup analysis using the CYP1A2 gene was performed to analyze the association between coffee and various disease genotypes. Likelihood ratio tests were used to assess the interactions between the CYP1A2 gene and coffee intake [4]. We also aimed to explore whether different coffee types had different associations with neurodegenerative diseases. We categorized participants into three groups according to daily coffee preference: ground coffee type, instant coffee type, and decaffeinated coffee type. The subgroup analysis using coffee types was employed to explore the associations among three coffee types and neurodegenerative diseases [5]. We included the number of cigarettes smoked per day by participants as a new covariate in the analysis [6]. After including the healthy diet score as a new covariate in the model, we again performed the analyses [7]. We used phenotypic age acceleration to represent the degree of aging in participants so as to explore the associations between coffee or tea intake and aging. RCS was used to explore the nonlinear association between coffee or tea intake and phenotypic age acceleration [8]. We reanalyzed the association between coffee or tea intake and neurodegenerative diseases after retaining participants with missing genetic data [9]. A reanalysis was performed by incorporating a history of coronary heart disease, diabetes, and cancer into a new model to explore the influence of a specific disease on the relationship between coffee or tea intake and neurodegenerative diseases [10]. To analyze the individual association between coffee and tea intake and neurodegenerative diseases, we did not manually adjust coffee and tea intake, allowing an assessment of their impact on the conclusions [11]. We included sugar and milk, two common dietary confounders, in the model for reanalysis to explore their influence on the results of coffee or tea consumption [12]. The subgroup analysis using hypertension and diabetes was used to explore the differences in the relationship between coffee or tea intake and neurodegenerative diseases between normal and diseased populations.

All statistical analyses in this study were performed using R software version 4.2.3. A two-sided *P*-value < 0.05 indicated a statistically significant difference.

## Results

### Characteristics of the study participants

This study included 134,425 individuals with a median follow-up of 13.5 years. During follow-up, 6483 participants developed neurodegenerative diseases, 4960 developed primary neurodegenerative diseases, 1026 developed vascular neurodegenerative diseases, 2556 developed other neurodegenerative diseases, 1964 developed AD, 1896 developed PD, 989 developed VD, and 254 developed ALS.

The baseline data for participants based on their coffee and tea intake are shown in Table 1. Participants who consumed more than five cups of coffee were mainly men, recent smokers, and recent drinkers with fewer hours of sleep and higher levels of CYP1A2 for AA compared with those who never consumed coffee. Those who drank more than three cups of tea compared with those who never drank tea were mainly men, more recent drinkers, less recent smokers and higher levels of milk addition when drinking coffee and tea. (Table 1).

**Table 1:** Baseline characteristics of study population according to the coffee consumption and tea consumption a)

a）					
Characteristics	Overall(*N* = 134425)	Coffee consumption			*P*-value^1^
		0(*N* = 23842)	0.5-5(*N* = 103489)	> 5(*N* = 7094)	
Age (mean (SD))	64.02 (2.83)	63.97 (2.86)	64.05 (2.83)	63.83 (2.81)	< 0.001
Sex = Male (%)	70,943 (52.8)	12,282 (51.5)	54,608 (52.8)	4053 (57.1)	< 0.001
Smoking status (%)					< 0.001
Never	65,401 (48.7)	11,852 (49.7)	51,039 (49.3)	2510 (35.4)	
Previous	58,301 (43.4)	10,164 (42.6)	44,899 (43.4)	3238 (45.6)	
Current	10,723 ( 8.0)	1826 ( 7.7)	7551 ( 7.3)	1346 (19.0)	
Drinking status (%)					< 0.001
Never	4820 ( 3.6)	1639 ( 6.9)	2913 ( 2.8)	268 ( 3.8)	
Previous	4467 ( 3.3)	1410 ( 5.9)	2708 ( 2.6)	349 ( 4.9)	
Current	125,138 (93.1)	20,793 (87.2)	97,868 (94.6)	6477 (91.3)	
APOE4 (%)					0.415
0	96,636 (71.9)	17,256 (72.4)	74,283 (71.8)	5097 (71.8)	
1	34,649 (25.8)	6050 (25.4)	26,774 (25.9)	1825 (25.7)	
2	3140 ( 2.3)	536 ( 2.2)	2432 ( 2.4)	172 ( 2.4)	
Sleep duration (%)					< 0.001
< 7	32,104 (23.9)	6214 (26.1)	23,879 (23.1)	2011 (28.3)	
7–8	92,000 (68.4)	15,604 (65.4)	71,863 (69.4)	4533 (63.9)	
> 8	10,321 ( 7.7)	2024 ( 8.5)	7747 ( 7.5)	550 ( 7.8)	
Education (%)					< 0.001
Lower secondary	21,013 (15.6)	3784 (15.9)	16,140 (15.6)	1089 (15.4)	
Upper secondary	6652 ( 4.9)	1023 ( 4.3)	5290 ( 5.1)	339 ( 4.8)	
Higher	76,906 (57.2)	11,549 (48.4)	61,458 (59.4)	3899 (55.0)	
Other	29,854 (22.2)	7486 (31.4)	20,601 (19.9)	1767 (24.9)	
BMI (%)					< 0.001
< 18.5	520 ( 0.4)	125 ( 0.5)	368 ( 0.4)	27 ( 0.4)	
18.5 to < 25	40,322 (30.0)	6983 (29.3)	31,699 (30.6)	1640 (23.1)	
25 to < 30	61,954 (46.1)	10,683 (44.8)	48,013 (46.4)	3258 (45.9)	
30 to < 35	23,857 (17.7)	4471 (18.8)	17,833 (17.2)	1553 (21.9)	
>=35	7772 ( 5.8)	1580 ( 6.6)	5576 ( 5.4)	616 ( 8.7)	
Stroke history = Yes (%)	2835 ( 2.1)	627 ( 2.6)	2042 ( 2.0)	166 ( 2.3)	< 0.001
Depression history = Yes (%)	6083 ( 4.5)	1287 ( 5.4)	4381 ( 4.2)	415 ( 5.9)	< 0.001
Diabetes history = Yes (%)	8684 ( 6.5)	1691 ( 7.1)	6403 ( 6.2)	590 ( 8.3)	< 0.001
Hypertension history = Yes (%)	44,171 (32.9)	8385 (35.2)	33,510 (32.4)	2276 (32.1)	< 0.001
Cancer history = Yes (%)	14,283 (10.6)	2585 (10.8)	10,989 (10.6)	709 (10.0)	0.125
Coronary heart disease history = Yes (%)	11,775 (8.8)	2528 (10.6)	8559 (8.3)	688 (9.7)	< 0.001
Overall health status (%)					< 0.001
Excellent	22,898 (17.0)	3491 (14.6)	18,320 (17.7)	1087 (15.3)	
Good	80,815 (60.1)	13,626 (57.2)	63,121 (61.0)	4068 (57.3)	
Fair	26,121 (19.4)	5538 (23.2)	18,999 (18.4)	1584 (22.3)	
Poor	4591 ( 3.4)	1187 ( 5.0)	3049 ( 2.9)	355 ( 5.0)	
Household income (%)					< 0.001
Less than £18,000	42,711 (31.8)	9499 (39.8)	30,706 (29.7)	2506 (35.3)	
£18,000 to £30,999	44,241 (32.9)	7470 (31.3)	34,525 (33.4)	2246 (31.7)	
£31,000 to £51,999	29,407 (21.9)	4413 (18.5)	23,574 (22.8)	1420 (20.0)	
£52,000 to £100,000	14,578 (10.8)	2033 ( 8.5)	11,801 (11.4)	744 (10.5)	
Greater than £100,000	3488 ( 2.6)	427 ( 1.8)	2883 ( 2.8)	178 ( 2.5)	
Achieve the recommended physical activity level = Yes (%)	112,717 (83.9)	19,665 (82.5)	87,307 (84.4)	5745 (81.0)	< 0.001
CYP1A2= CC_AC (%)	63,399 (47.2)	11,554 (48.5)	48,644 (47.0)	3201 (45.1)	< 0.001
Phenotypic age acceleration^2^ (SD))	0.00 (5.80)	0.59 (6.19)	-0.18 (5.70)	0.65 (5.79)	< 0.001
Healthy diet score^2^ (%)					< 0.001
0	2219 ( 1.7)	479 ( 2.1)	1545 ( 1.5)	195 ( 2.8)	
1	11,223 ( 8.5)	2235 ( 9.6)	8166 ( 8.0)	822 (11.8)	
2	25,550 (19.4)	4653 (20.0)	19,270 (19.0)	1627 (23.5)	
3	35,873 (27.2)	6263 (26.9)	27,658 (27.2)	1952 (28.1)	
4	35,627 (27.0)	6000 (25.8)	28,094 (27.6)	1533 (22.1)	
5	21,411 (16.2)	3663 (15.7)	16,939 (16.7)	809 (11.7)	
Number of cigarettes smoked per day (mean (SD))	0.80 (3.92)	0.88 (4.16)	0.66 (3.51)	2.61 (7.11)	< 0.001
Sugar addition **=** Yes (%)	3283 ( 2.4)	66 ( 0.3)	3036 ( 2.9)	181 (2.6)	< 0.001
Milk addition = Yes (%)	14,792 (11.0)	2472 (10.4)	11,865 (11.5)	455 (6.4)	< 0.001

b)					
Characteristics	Overall(N=134425)	Tea consumption			P-value^1^
		0(N=17211)	0.5-3(N=54966)	>3(N=62248)	
Age (mean (SD))	64.02 (2.83)	63.88 (2.81)	64.03 (2.83)	64.06 (2.84)	<0.001
Sex = Male (%)	70943 (52.8)	8522 (49.5)	29399 (53.5)	33022 (53.0)	<0.001
Smoking status (%)					<0.001
Never	65401 (48.7)	7821 (45.4)	26808 (48.8)	30772 (49.4)	
Previous	58301 (43.4)	7543 (43.8)	24288 (44.2)	26470 (42.5)	
Current	10723 ( 8.0)	1847 (10.7)	3870 ( 7.0)	5006 ( 8.0)	
Drinking status (%)					<0.001
Never	4820 ( 3.6)	922 ( 5.4)	1455 ( 2.6)	2443 ( 3.9)	
Previous	4467 ( 3.3)	824 ( 4.8)	1380 ( 2.5)	2263 ( 3.6)	
Current	125138 (93.1)	15465 (89.9)	52131 (94.8)	57542 (92.4)	
APOE4 (%)					<0.001
0	32104 (23.9)	4556 (26.5)	12710 (23.1)	14838 (23.8)	
1	92000 (68.4)	11261 (65.4)	38223 (69.5)	42516 (68.3)	
2	10321 ( 7.7)	1394 ( 8.1)	4033 ( 7.3)	4894 ( 7.9)	
Sleep duration (%)					0.606
<7	96636 (71.9)	12368 (71.9)	39557 (72.0)	44711 (71.8)	
7-8	34649 (25.8)	4438 (25.8)	14091 (25.6)	16120 (25.9)	
>8	3140 ( 2.3)	405 ( 2.4)	1318 ( 2.4)	1417 ( 2.3)	
Education (%)					<0.001
Lower secondary	21013 (15.6)	2800 (16.3)	8523 (15.5)	9690 (15.6)	
Upper secondary	6652 ( 4.9)	937 ( 5.4)	3004 ( 5.5)	2711 ( 4.4)	
Higher	76906 (57.2)	9613 (55.9)	33353 (60.7)	33940 (54.5)	
Other	29854 (22.2)	3861 (22.4)	10086 (18.3)	15907 (25.6)	
BMI (%)					<0.001
<18.5	520 ( 0.4)	76 ( 0.4)	212 ( 0.4)	232 ( 0.4)	
18.5 to <25	40322 (30.0)	4533 (26.3)	17179 (31.3)	18610 (29.9)	
25 to <30	61954 (46.1)	7462 (43.4)	25274 (46.0)	29218 (46.9)	
30 to <35	23857 (17.7)	3633 (21.1)	9385 (17.1)	10839 (17.4)	
>=35	7772 ( 5.8)	1507 ( 8.8)	2916 ( 5.3)	3349 ( 5.4)	
Stroke history = Yes (%)	2835 ( 2.1)	450 ( 2.6)	1054 ( 1.9)	1331 ( 2.1)	<0.001
Depression history = Yes (%)	6083 ( 4.5)	824 ( 4.8)	2268 ( 4.1)	2991 ( 4.8)	<0.001
Diabetes history = Yes (%)	8684 ( 6.5)	1492 ( 8.7)	3451 ( 6.3)	3741 ( 6.0)	<0.001
Hypertension history = Yes (%)	44171 (32.9)	5745 (33.4)	17959 (32.7)	20467 (32.9)	0.224
Cancer history = Yes (%)	14283 (10.6)	1801 (10.5)	5746 (10.5)	6736 (10.8)	0.096
Coronary heart disease history = Yes (%)	11775 (8.8)	1664 (9.7)	4422 (8.0)	5689 (9.1)	<0.001
Overall health status (%)					<0.001
Excellent	22898 (17.0)	2947 (17.1)	9910 (18.0)	10041 (16.1)	
Good	80815 (60.1)	9924 (57.7)	33664 (61.2)	37227 (59.8)	
Fair	26121 (19.4)	3562 (20.7)	9828 (17.9)	12731 (20.5)	
Poor	4591 ( 3.4)	778 ( 4.5)	1564 ( 2.8)	2249 ( 3.6)	
Household income (%)					<0.001
Less than £18,000	42711 (31.8)	5739 (33.3)	15128 (27.5)	21844 (35.1)	
£18,000 to £30,999	44241 (32.9)	5597 (32.5)	18085 (32.9)	20559 (33.0)	
£31,000 to £51,999	29407 (21.9)	3626 (21.1)	12965 (23.6)	12816 (20.6)	
£52,000 to £100,000	14578 (10.8)	1828 (10.6)	6906 (12.6)	5844 ( 9.4)	
Greater than £100,000	3488 ( 2.6)	421 ( 2.4)	1882 ( 3.4)	1185 ( 1.9)	
Achieve the recommended physical activity level= Yes (%)	112717 (83.9)	14039 (81.6)	46057 (83.8)	52621 (84.5)	<0.001
CYP1A2= CC_AC (%)	63399 (47.2)	8098 (47.1)	26446 (48.1)	28855 (46.4)	<0.001
Phenotypic age acceleration^2^ (SD))	0.00 (5.80)	0.35 (6.15)	-0.22 (5.70)	0.10 (5.79)	<0.001
Healthy diet score^2^ (%)					<0.001
0	2219 ( 1.7)	371 ( 2.2)	835 ( 1.5)	1013 ( 1.7)	
1	11223 ( 8.5)	1625 ( 9.6)	4378 ( 8.1)	5220 ( 8.6)	
2	25550 (19.4)	3453 (20.5)	10207 (18.9)	11890 (19.5)	
3	35873 (27.2)	4697 (27.8)	14494 (26.8)	16682 (27.4)	
4	35627 (27.0)	4264 (25.3)	15015 (27.8)	16348 (26.8)	
5	21411 (16.2)	2469 (14.6)	9114 (16.9)	9828 (16.1)	
Number of cigarettes smoked per day (mean (SD))	0.80 (3.92)	1.30 (5.10)	0.60 (3.33)	0.85 (4.01)	<0.001
Sugar addition= Yes (%)	3283 ( 2.4)	432 (2.5)	1510 ( 2.7)	1341 ( 2.2)	<0.001
Milk addition= Yes (%)	14792 (11.0)	404 (2.3)	6319 (11.5)	8069 (13.0)	<0.001

a) study population segmented by using coffee intake and b) study population segmented using tea intake

^1^Analysis of variance or χ2 test where appropriate

^2^The number of participants with healthy meal scores was 131,903 and the number of participants with Phenotypic age acceleration was 113,993

### Nonlinear association of coffee or tea intake with neurodegenerative diseases

**Fig. 2:**
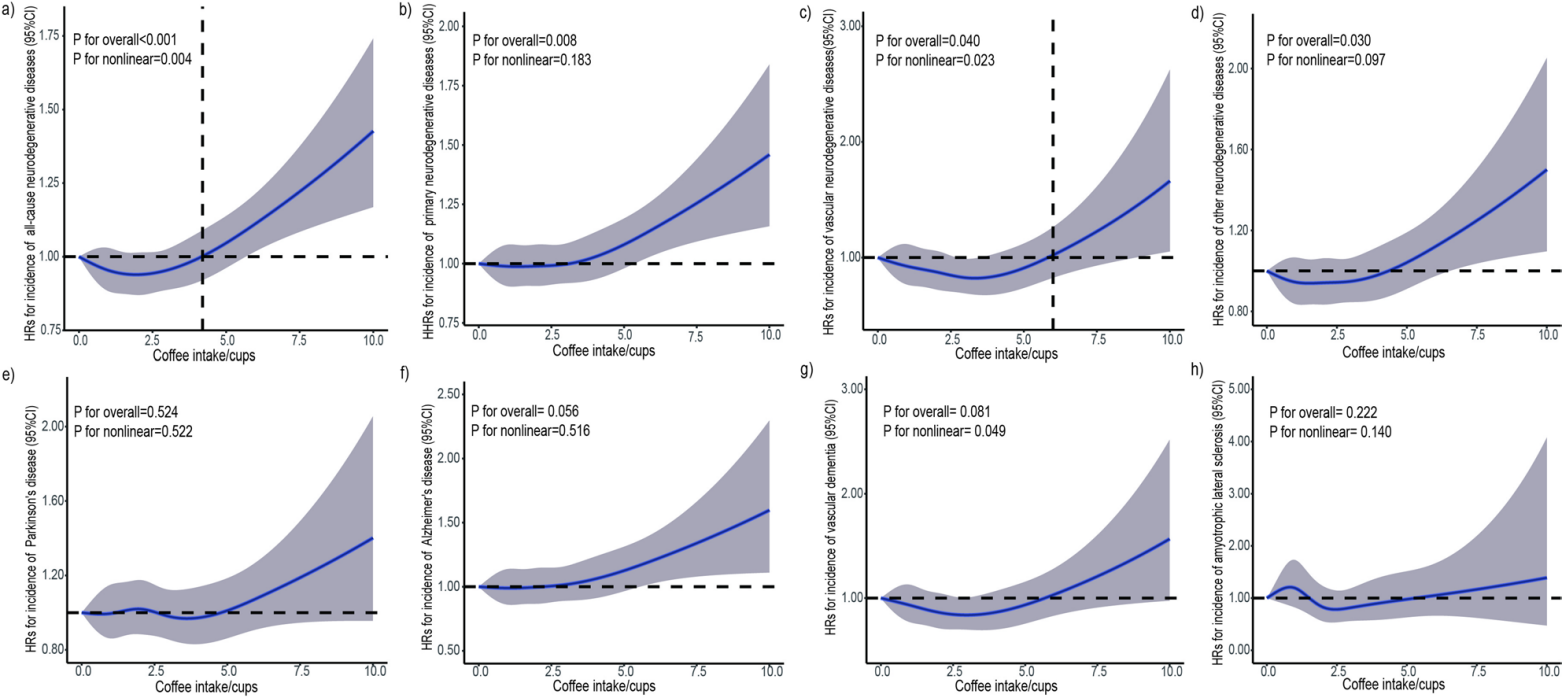
The nonlinear association of coffee consumption with the risk of incident neurodegenerative disease.Restricted cubic spline for testing the hypothesis of nonlinear correlation between (**a**) all-cause neurodegenerative disease, (**b**) primary neurodegenerative disease, (**c**) vascular neurodegenerative disease, (**d**) other neurodegenerative disease, (**e**) Parkinson’s disease, (**f**) Alzheimer's disease, (**g**) vascular dementia, (**h**) amyotrophic lateral sclerosis, and coffee intake. Spline curves represent hazard ratios (HRs) adjusted for age, sex, smoking statue, drinking statue, sleep duration, education, BMI, stroke history, depression history, overall health status, income, physical activity, CYP1A2 and tea intake. And in assessing the relationship between Alzheimer's disease and coffee intake, the APOE4 gene is additionally adjusted. The solid lines are fitted based on Cox proportional-hazard models. The shaded areas show 95% confidential intervals (CIs)

We used RCS to investigate the nonlinear association between coffee or tea intake and neurodegenerative diseases. A significant nonlinear association was found between coffee intake and all-cause neurodegenerative diseases (*P* for overall < 0.001, *P* for nonlinear = 0.004) as well as vascular neurodegenerative diseases (*P* for overall = 0.040, *P* for nonlinear = 0.023) (Fig. 2). Both curves were J-shaped. It suggested that excessive amounts of coffee were associated with an increased risk of all-cause neurodegenerative diseases and vascular neurodegenerative diseases (> 4 cups for all-cause neurodegenerative diseases and > 6 cups for vascular neurodegenerative diseases). Coffee intake was significantly correlated with primary neurodegenerative diseases (*P* for overall = 0.008, *P* for nonlinear = 0.183) and other neurodegenerative diseases (*P* for overall = 0.030, *P* for nonlinear = 0.097), but with no significant nonlinear association. Also, no significant association was observed between PD (*P* for overall = 0.524, *P* for nonlinear = 0.522), AD (*P* for overall = 0.056, *P* for nonlinear = 0.516), VD (*P* for overall = 0.081, *P* for nonlinear = 0.049), and ALS (*P* for overall = 0.222, *P* for nonlinear = 0.140).

**Fig. 3:**
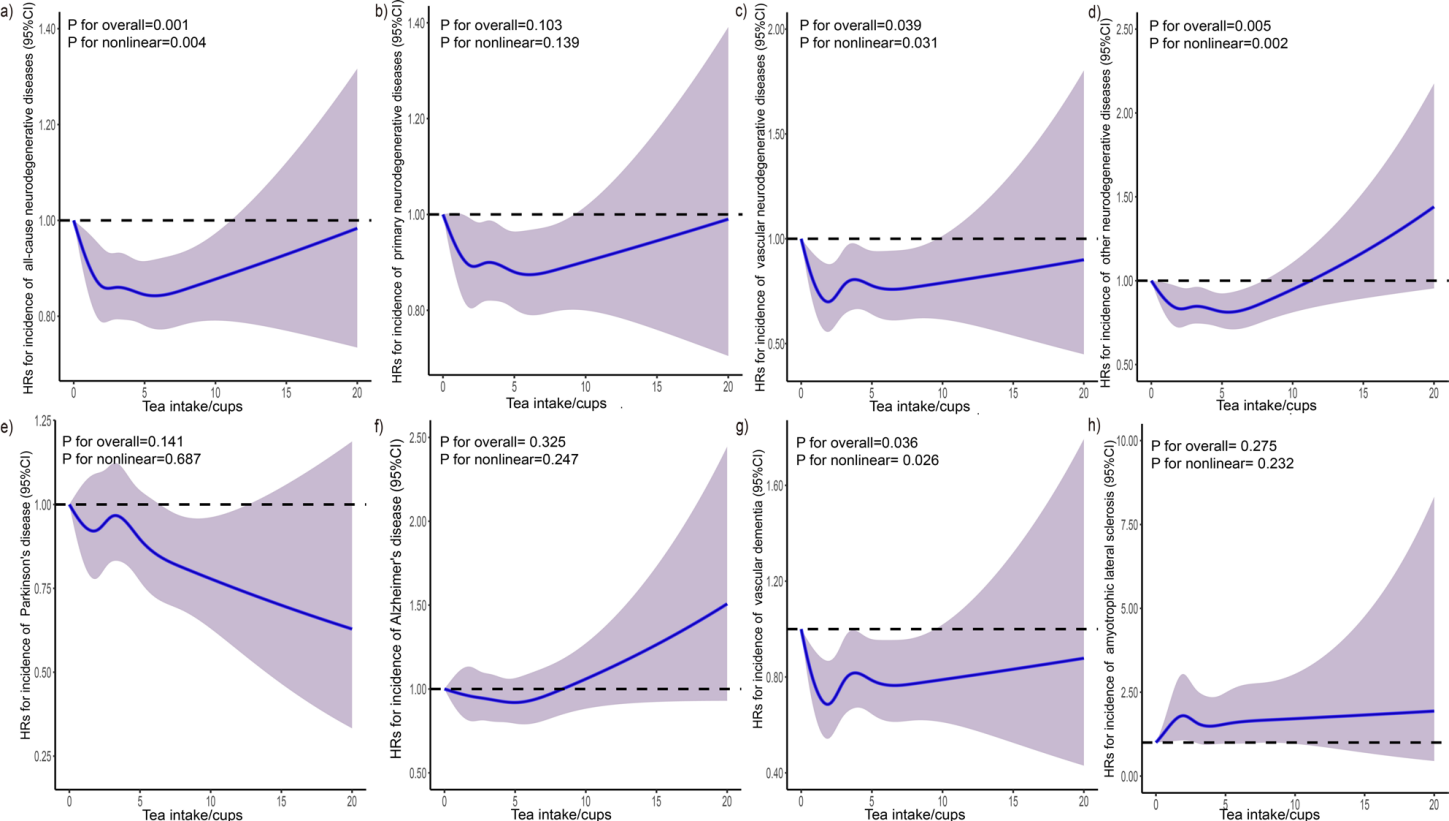
The nonlinear association of tea consumption with the risk of incident neurodegenerative diseaseRestricted cubic spline for testing the hypothesis of nonlinear correlation between (**a**) all-cause neurodegenerative disease, (**b**) primary neurodegenerative disease, (**c**) vascular neurodegenerative disease, (**d**) other neurodegenerative disease, (**e**) Parkinson’s disease, (**f**) Alzheimer's disease, (**g**) vascular dementia, (**h**) amyotrophic lateral sclerosis, and tea intake. Spline curves represent hazard ratios (HRs) adjusted for age, sex, smoking statue, drinking statue, sleep duration, education, BMI, stroke history, depression history, overall health status, income, physical activity, CYP1A2 and coffee intake. And in assessing the relationship between Alzheimer's disease and tea intake, the APOE4 gene is additionally adjusted. The solid lines are fitted based on Cox proportional-hazard models. The shaded areas show 95% confidential intervals (CIs)

Further, a significant nonlinear association was noted between tea intake and all-cause neurodegenerative diseases (*P*-value = 0.001, *P* for nonlinear = 0.004), vascular neurodegenerative diseases (*P*-value = 0.039, *P* for nonlinear = 0.031), other neurodegenerative diseases (*P*-value = 0.005, *P* for nonlinear = 0.002), and VD (*P*-value = 0.036, *P* for nonlinear = 0.026). The curve was approximately U-shaped (Fig. 3). No significant nonlinear associations were observed for primary neurodegenerative diseases (*P*-value = 0.103, *P* for nonlinear = 0.139), PD (*P*-value = 0.141, *P* for nonlinear = 0.687), AD (*P*-value = 0.325, *P* for nonlinear = 0.247), and ALS (*P*-value = 0.275, *P* for nonlinear = 0.232) (Fig. 3).

### Individual association between coffee or tea and neurodegenerative diseases

We divided the participants based on the cups of coffee consumed into three groups to reassess the association between coffee and tea and neurodegenerative diseases: 0, 0.5–5, and > 5. The participants were divided into three groups based on the cups of tea consumed: 0, 0.5–3, and > 3. We reassessed the relationship between coffee or tea intake and neurodegenerative diseases and their subtypes using the Cox proportional-hazards regression model. For primary neurodegenerative diseases (HR = 1.16, 95% CI = 1.01–1.33, P-value = 0.034), the risk of morbidity was higher in participants consuming > 5 cups of coffee per day compared with those who never drink coffee. For all-cause neurodegenerative diseases (HR = 1.11, 95% CI = 0.99–1.25, P-value = 0.074) and AD (HR = 1.23, 95% CI = 0.99–1.52, P-value = 0.06), a slightly significant difference in the risk of morbidity was observed for those who consumed more than five cups of coffee per day compared with those who never drank coffee.

**Fig. 4:**
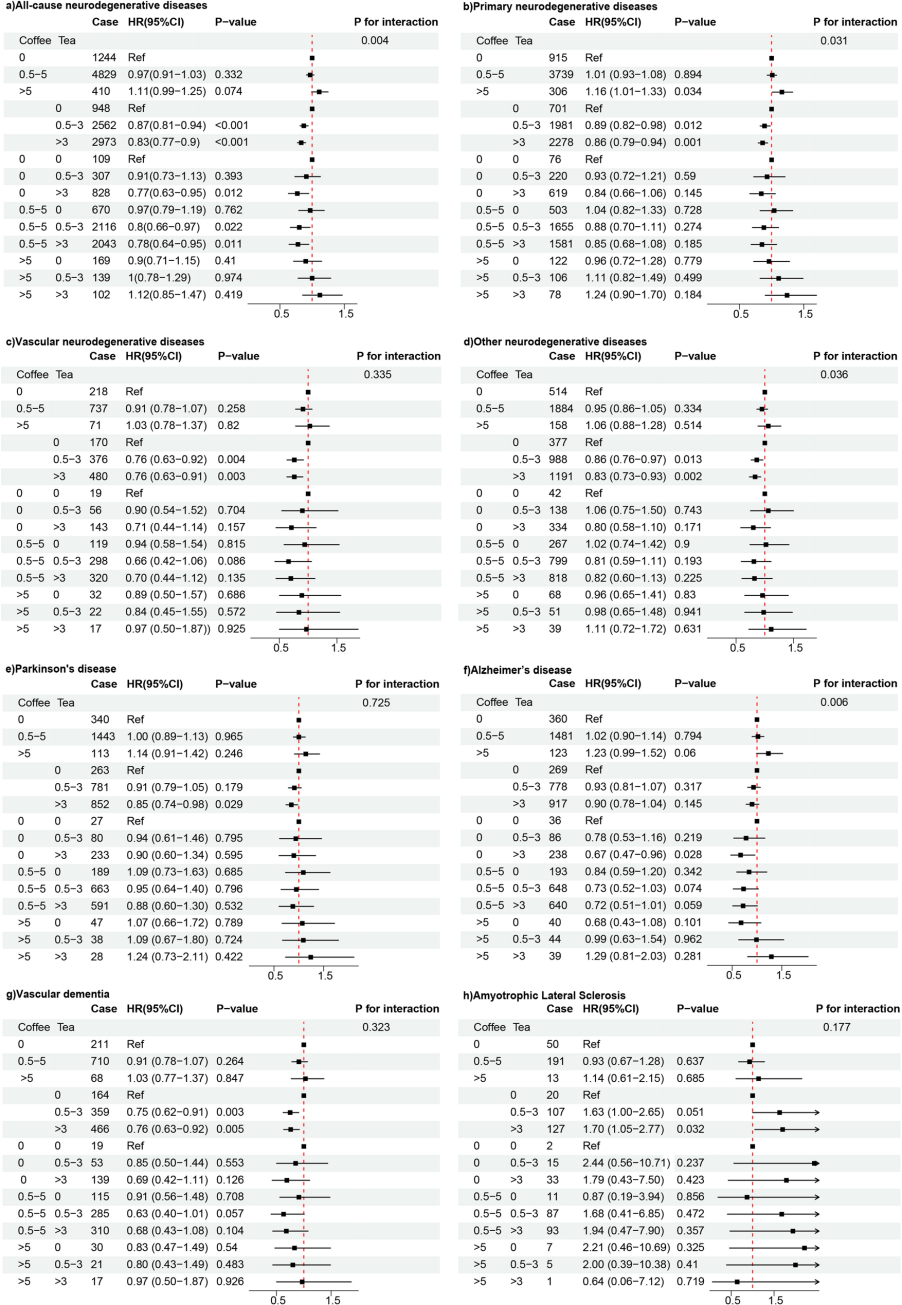
Association of coffee and tea intake with the risk of incident neurodegenerative diseaseCoffee and tea intake with the risk of incident (**a**) all-cause neurodegenerative disease, (**b**) primary neurodegenerative disease, (**c**) vascular neurodegenerative disease, (**d**) other neurodegenerative disease, (**e**) Parkinson’s disease, (**f**) Alzheimer's disease, (**g**) vascular dementia, (**h**) amyotrophic lateral sclerosis. Multivariable model is adjusted for age, sex, smoking statue, drinking statue, sleep duration, education, BMI, stroke history, depression history, overall health status, income, physical activity, CYP1A2. And in assessing the relationship between Alzheimer's disease and coffee or tea intake, the APOE4 gene is additionally adjusted. The coffee and tea intake will be mutually adjusted

In addition, the risk of all-cause neurodegenerative diseases (HR = 0.87, 95% CI = 0.81–0.94, *P*-value < 0.001), primary neurodegenerative diseases (HR = 0.89, 95% CI = 0.82–0.98, *P*-value = 0.012), vascular neurodegenerative diseases (HR = 0.76, 95% CI = 0.63–0.92, *P*-value = 0.004), other neurodegenerative diseases (HR = 0.86, 95% CI = 0.76–0.97, *P*-value = 0.013), and VD (HR = 0.75, 95% CI = 0.62–0.91, *P*-value = 0.003) was lower with a daily intake of 0.5–3 cups of tea compared with those who never drank tea. Similar protective associations were observed on all-cause neurodegenerative diseases (HR = 0.83, 95% CI = 0.77–0.90, *P*-value < 0.001), primary neurodegenerative diseases (HR = 0.86, 95% CI = 0.79–0.94, *P*-value < 0.001), vascular neurodegenerative diseases (HR = 0.76, 95% CI = 0.63–0.91, *P*-value = 0.003), other neurodegenerative diseases (HR = 0.83, 95% CI = 0.73–0.93, *P*-value = 0.002), PD (HR = 0.85, 95% CI = 0.74–0.98, *P*-value = 0.029), and VD (HR = 0.76, 95% CI = 0.63–0.92, *P*-value = 0.005) when consuming > 3 cups of tea per day compared with those who never drank tea. However, those who consumed 0.5–3 cups of tea per day (HR = 1.63, 95% CI = 1.00–2.65, *P*-value = 0.051) or > 3 cups (HR = 1.70, 95% CI = 1.05–2.77, *P*-value = 0.032) had an increased risk of ALS compared with those who never consumed tea (Fig. 4).

### Interactive association between coffee and tea intake and neurodegenerative diseases

We further investigated the interactive associations of coffee and tea intake on neurodegenerative diseases and their subtypes. A significant interaction was found between coffee and tea intake on all-cause neurodegenerative diseases (*P* for interaction = 0.004); 0 cups of coffee and > 3 cups of tea (HR = 0.77, 95% CI = 0.63–0.95, *P*-value = 0.012); 0.5–5 cups of coffee and 0.5–3 cups of tea (HR = 0.80, 95% CI = 0.66–0.97, *P*-value = 0.022), and 0.5–5 cups of coffee and > 3 cups of tea (HR = 0.78, 95% CI = 0.66–0.97, *P*-value = 0.022) were observed to be significantly associated with a reduced risk of all-cause neurodegenerative disease. Moreover, a significant interaction was also observed between tea and coffee intake on AD (*P* for interaction = 0.006). Those who consumed 0 cups of coffee and > 3 cups of tea per day (HR = 0.67, 95% CI = 0.47–0.96, *P*-value = 0.028) had a lower risk of morbidity compared with those who did not consume tea or coffee. A significant interaction was found between coffee and tea intake on primary (*P* for interaction = 0.031) and other neurodegenerative diseases (*P* for interaction = 0.036). However, no combination of coffee and tea showed a significant association with a reduced risk of other neurodegenerative diseases. In contrast, no significant interaction was noted between coffee and tea on vascular neurodegenerative diseases (*P* for interaction = 0.335), PD (*P* for interaction = 0.725), VD (*P* for interaction = 0.323), and ALS (*P* for interaction = 0.177) (Fig. 4).

### Sensitivity analysis

When using the coffee type subgroups, the relationships between different coffee types and all-cause neurodegenerative diseases (*P* for interaction = 0.018) were not identical (Fig. S2). Participants who consumed 0.5–5 cups of ground coffee per day (HR = 0.89, 95% CI = 0.80–0.97, *P*-value = 0.012) had a lower risk of all-cause neurodegenerative disease onset than those who never consumed coffee. A slightly significant interaction was also observed with primary neurodegenerative diseases (*P* for interaction = 0.057), where participants who consumed 0.5–5 cups of decaffeinated coffee per day had a higher risk of morbidity compared with those who never drank coffee (HR = 1.13, 95% CI = 1.03–1.25, *P*-value = 0.011). No significant differences in the association between different coffee types and vascular neurodegenerative diseases, other neurodegenerative diseases, PD, AD, VD, and ALS were observed. For all-cause neurodegenerative diseases (*P* for interaction = 0.791), primary neurodegenerative diseases (*P* for interaction = 0.605), vascular neurodegenerative diseases (*P* for interaction = 0.376), other neurodegenerative diseases (*P* for interaction = 0.420), PD (*P* for interaction = 0.356), AD (*P* for interaction = 0.953), VD (*P* for interaction = 0.579), and ALS (*P* for interaction = 0.447), the *P*-values for the interaction between coffee and the *CYP1A2* gene were all greater than 0.05. This suggested no significant interaction between the *CYP1A2* gene and coffee intake (Fig. S3). After excluding the number of people with a prevalence of less than 2 years, the multivariate Cox proportional-hazards model was reused to explore the association among coffee or tea and neurodegeneration, and the results were similar to those of the main analysis (Fig. S4). When the RCS model with four knots was used, the results were similar to those of the RCS model with five knots (Figs. S5 and S6). After additional adjustments for the number of cigarettes smoked per day by participants, the analysis results were consistent with previous findings (Fig. S7). After adjusting for dietary variables, the results were consistent with the main analysis findings (Fig. S8). A significant U-shaped association was observed between coffee intake and phenotypic age acceleration (*P* for interaction < 0.001). A significant nonlinear association was also observed between tea intake and phenotypic age acceleration (*P* for interaction < 0.001) (Fig. S9). After retaining the participants with missing genes, the analysis results were similar to the primary analysis results (Fig. S10). When three disease histories were added to the new model, the results of the analysis did not deviate significantly from the previous results (Fig. S11). Without mutual adjustment for coffee and tea intake, the significance of the results did not change (Table S2). The results remained consistent after adjusting for two variables, milk and sugar, confirming that milk and sugar addition did not influence the relationship between coffee or tea intake with neurodegenerative diseases (Fig. S12). The subgroup analyses revealed significant differences in the association between coffee intake and all-cause neurodegenerative diseases between healthy and hypertensive populations (*P* for interaction = 0.020). In the hypertensive population, participants who consumed > 5 cups of coffee per day had a significantly higher risk of developing all-cause neurodegenerative diseases compared with non-coffee drinkers (HR = 1.31, 95% CI = 1.09–1.57, *P*-value = 0.004). Also, a slightly significant interaction was observed between coffee intake and diabetes in ALS (*P* for interaction = 0.058) (Table S3).

## Discussion

Our large cohort longitudinal study showed that [1] excessive coffee intake was associated with the increased risk of primary neurodegenerative diseases and AD [2]. Moderate tea intake was significantly related to a reduced risk of all-cause neurodegenerative diseases, vascular neurodegenerative diseases, other neurodegenerative diseases, VD and PD [3]. Coffee and tea intake had interactive associations on all-cause neurodegenerative diseases and AD [4]. Coffee variety modulated the association between coffee intake and all-cause neurodegenerative diseases and primary neurodegenerative diseases [5]. No significant interactive association between coffee intake and the CYP1A2 gene was found on the risk of all-cause neurodegenerative diseases and their subtypes, suggesting that the CYP1A2 gene might not influence association between coffee intake and neurodegenerative diseases [6]. The relationship of coffee intake and all-cause neurodegenerative diseases differed in normal and hypertensive populations.

A meta-analysis showed that moderate coffee intake is associated with a reduced risk of developing dementia [27]. Another study using data from the Italian Longitudinal Study on Aging suggested that a small amount of coffee intake lowered the likelihood of cognitive deterioration in elderly individuals. In contrast, excessive coffee intake increased the risk of cognitive decline [28]. This was partially consistent with our results. A longitudinal cohort of the UK Biobank–based study suggested that individuals who consumed excessive coffee per day (six cups and more) were at a higher risk of developing dementia than those with lesser coffee intake (one to two cups/day) [8]. Another study reported no significant correlation between coffee intake and PD [29], consistent with the results of this study. Despite a nonlinear association between coffee intake and all-cause neurodegenerative diseases, no significant association was observed between coffee and AD. However, after subgrouping coffee intake to include it in the Cox proportional-hazards model, those who consumed more than six cups of coffee per day had a higher risk of developing AD than those who never consumed coffee. A Mendelian randomization study also found that higher coffee consumption was statistically significantly correlated with a higher risk of AD, which was similar to our conclusion [30]. For VD, a significant association with coffee intake was observed using RCS with four knots, but a less significant association was noted using RCS with five knots. This was probably because the small number of patients with VD in this study led to unstable results. Hence, follow-up studies with larger samples of VD are needed to validate the association between coffee intake and VD.

One possible reason why moderate amounts of coffee is associated with a reduced risk of neurodegenerative diseases is that the caffeine in coffee affects neuronal plasticity and resilience, thereby protecting the brain and reducing the risk of neurodegenerative diseases [6]. Another possible mechanism by which moderate coffee intake may reduce neurodegenerative diseases is that coffee contains polyphenols with anti-inflammatory and antioxidant properties, which may help prevent neurodegeneration and cognitive decline [31]. One possible explanation for the increased risk of neurodegenerative diseases associated with excessive coffee intake is that coffee intake leads to increased serum total cholesterol, low-density lipoprotein cholesterol, and triglyceride levels [32]. These lipids are major risk factors for atherosclerosis, increasing the risk of AD and vascular neurodegenerative diseases [33]. Another possible explanation is that excessive coffee intake causes the shrinkage of gray matter in the brain. The more the brain volume decreases and the weaker the brain functions, the more likely it is to cause neurodegenerative diseases. The outcomes of a randomized, double-blind, controlled trial indicated that the experimental group consuming caffeine for a week significantly decreased gray matter volume and had a lower score on the working memory test compared with the placebo control group [34]. Another longitudinal cohort study based on the UK Biobank also found a significant decrease in the total brain and gray matter volumes with excessive coffee intake [8].

Some recent studies showed a statistically significant correlation between tea intake and a lower risk of developing PD [35, 36]. Another study on Chinese and Japanese people showed a significant negative correlation between green tea intake and the risk of PD [36]. Meanwhile, a UK Biobank–based cohort study demonstrated that consuming tea alone or both tea and coffee was associated with a lower incidence of dementia [9]. This was similar to our findings. Some studies found no significant association between tea intake and ALS [37, 38]. However, some studies supported the idea that tea intake might reduce the risk of ALS onset [39]. This was inconsistent with our conclusions. It might be due to the small number of patients with ALS included in the aforementioned studies and our study, thus creating a bias. Subsequent analyses with a larger number of patients with ALS need to be performed to determine the actual relationship among tea intake and ALS.

Possible reasons for linking tea consumption to a reduced incidence of neurodegenerative diseases include the presence of catechin, which is a phenolic compound present in tea. Oxidative stress has been reported as a key mechanism underlying the development of neurodegenerative diseases [40]. Catechins serve as antioxidants in the body, thereby reducing the incidence of neurodegenerative diseases. The possible mechanism underlying the antioxidant activity of catechins in the body is that catechins chelate divalent metal ions and prevent oxidation induced by reactive hydroxyl radicals [41]. Second, amyloid-β aggregation may be one of the main mechanisms underlying the development of dementia [42]. Tea contains epigallocatechin gallate, an active component of catechins, which can inhibit amyloid-β aggregation and thus help moderate the progression of neurodegenerative diseases [43]. Third, animal studies demonstrated that green tea polyphenols might protect dopamine neurons by inhibiting the production of nitric oxide and reactive oxygen species, thereby reducing the risk of neurodegeneration [44].

We found a significant interactive association between coffee and tea intake and all-cause neurodegenerative diseases and AD. Also, recent evidence indicates a interactive association between coffee and tea intake and some diseases, such as dementia [9]. The following mechanisms may account for the interactive association between coffee and tea intake and all-cause neurodegenerative diseases and AD. Coffee and tea share common components such as caffeine. Moderate amounts of caffeine have a protective effect on the brain and may reduce the risk of neurodegenerative diseases associated with aging [6]. However, coffee and tea do not contain the same amounts of various components. For example, the main phenolics in tea are catechins, whereas those in coffee are hydroxycinnamic acids. Both hydroxycinnamic acids and catechins have anti-inflammatory and antioxidant effects, and they have different target molecules [45]. Studies have indicated a direct antioxidant effect of catechins by chelating free transition metals such as iron and copper [46]. However, one possible explanation for the protective effect of hydroxycinnamic acids is their ability to inhibit the activity of 5-lipoxygenase, an enzyme whose activation is associated with cellular damage [47, 48]. Thus, the specific polyphenol contents of coffee and tea may play a combined protective role in the pathogenesis of neurodegenerative diseases due to their different target molecules. Another possible mechanism is that coffee and tea intake may jointly regulate the activation of certain cytokines [49–51]. The potential interactive association between coffee and tea intake and neurodegenerative diseases in animal studies need further validation.

Our results found that coffee type modulated the associations between coffee intake and neurodegenerative diseases. Only a moderate intake of ground coffee, among the three coffee types, was associated with a decreased risk of all-cause neurodegenerative diseases. One possible explanation for this reduction is the neuroprotective effect of chlorogenic acid found in ground coffee [52]. A previous study demonstrated that ground coffee had the highest levels of chlorogenic acids among 83 various coffee-related products [53]. Also, only decaffeinated coffee influenced the development of primary neurodegenerative diseases. A recent study suggested that moderate intake of ground coffee might reduce the risk of dementia, and decaffeinated coffee might be associated with a higher risk of developing AD [25]. These findings aligned with the conclusions of our study. The reasons why decaffeinated coffee is associated to the increasing risk of primary neurodegenerative diseases need to be explored in subsequent studies.

Our results indicated no significant interaction between the *CYP1A2* gene and coffee intake for various neurodegenerative disorders, suggesting that coffee played a similar role for neurodegenerative diseases in the *CYP1A2* gene subtype. A study using three large prospective cohorts, including the Nurses’ Health Study, concluded that the *CYP1A2* gene did not modulate the association between coffee intake and PD [54]. Also, a randomized controlled study showed that the *CYP1A2* gene did not modulate the link among coffee and the cognitive level of visual attention [55, 56]. In addition, a genetic caffeine metabolism score (CMS) was calculated using the UK Biobank data with two single-nucleotide polymorphisms (rs2472297 and rs6968554) associated with caffeine metabolism [57]. Higher scores indicated greater metabolic capacity. The results of the aforementioned study suggested no significant interaction between CMS and coffee intake [58]. These findings were similar to our study results. However, a survey of older French adults showed that the *CYP1A2* gene modulated the relationship between coffee intake and dementia; a linear association between coffee intake and dementia was observed only in participants who metabolized coffee more slowly but not in those who metabolized coffee more quickly [59].

Our results showed that the relationship between coffee intake and all-cause neurodegenerative diseases varied between normal and hypertensive populations. The risk of developing all-cause neurodegenerative diseases in patients with hypertension was significantly higher in participants who consumed > 5 cups of coffee per day compared with those who did not drink coffee. Further, patients with hypertension may be more sensitive to caffeine, which may lead to elevated blood pressure [60, 61], thereby increasing the risk of developing all-cause neurodegenerative diseases. A significant interaction between coffee and diabetes was also observed in ALS, possibly due to bias resulting from an extremely small number of patients with ALS in the study. Further research is needed to validate these findings.

Our study had the following advantages (1). We not only investigated the individual association between coffee or tea intake and neurodegenerative diseases but also explored the interactive association between coffee and tea intake and neurodegenerative diseases (2). This study investigated whether the CYP1A2 gene modulated the relationship among coffee intake and neurodegenerative diseases by influencing caffeine metabolism in the body (3). This study examined the association between coffee intake and the incidence of all-cause neurodegenerative diseases and multiple subtypes of neurodegenerative diseases, filling the gap of previous studies exploring the link between coffee intake and only a single type of neurodegenerative disease. However, our study also had certain limitations. First, the coffee and tea intake details were filled in based on the participants’ recollections of the intake in the past, leading to some errors. Follow-up studies should be conducted by periodically reminding participants to keep track of their coffee and tea intake rather than relying on later recall. Alternatively, we should consider collecting data on the biomarkers of caffeine or polyphenols in urine or blood samples as objective indicators. Second, the individuals who participated in this study were all of European origin. Hence, more investigation is needed to determine whether the results can be generalized to various races. Third, this was an observational study. Hence, although covariates were adjusted and multiple sensitivity analyses were performed, certain unknown confounding factors might have interfered with the results. Further studies are needed to confirm the reliability of these findings. Finally, as this study was conducted in a single cohort, validation in other cohorts is needed to confirm the robustness of the results.

## Conclusions

The coffee intake had a J-shaped association with all-cause neurodegenerative diseases and vascular neurodegenerative diseases. The U-shaped associations were found between tea intake and all-cause neurodegenerative diseases, vascular neurodegenerative diseases, other neurodegenerative diseases, and VD. Also, coffee and tea intake had an interactive association with all-cause neurodegenerative diseases and AD. Moreover, coffee type modulated the association between coffee intake and all-cause neurodegenerative diseases and primary neurodegenerative diseases. Further, *CYP1A2*, a gene that regulated coffee metabolism, did not modulate the association between coffee intake and all-cause neurodegenerative diseases and their subtypes.

## Supplementary Information

Supplementary Material 1.

## Data Availability

Data obtained from the UK Biobank are available on application at https://www.ukbiobank.ac.uk/use-our-data/apply-for-access (Application Number: 94166).

## Abbreviations

AD: Alzheimer’s disease
ALS: amyotrophic lateral sclerosis
APOE e4: apolipoprotein E ε4
BMI: body mass index
CI: confidence interval
CMS: caffeine metabolism score
CYP1A2: cytochrome P450 Family 1 Subfamily A Member 2
HR: hazard ratio
ICD: Classification of Diseases
PD: Parkinson’s disease
RCS: restricted cubic spline
VD: vascular dementia
