# Supplementary material for: Consumption of coffee and tea and the risk of developing neurodegenerative diseases: a cohort study in the UK biobank
Source: Nutr J. 2026 Mar 3;25:39. doi: 10.1186/s12937-026-01291-0 (PMC13064356; doi:10.1186/s12937-026-01291-0)
Supplement: Supplementary file 1 — Supplementary Material 1. [file 12937_2026_1291_MOESM1_ESM.docx]

**Fig. S1 The structural breakpoint of coffee intake and tea intake**

The structural breakpoint of coffee intake and b) The structural breakpoint of tea intake. The figure is based on the Cox proportional-hazards model. And the model is adjusted for age, sex, smoking statue, drinking statue, sleep duration, education, BMI, stroke history, depression history, overall health status, income, physical activity, CYP1A2. And coffee and tea intake will be mutually adjusted. The yellow line fitted the HR curve for each coffee or tea intake compared to those who did not consume coffee or tea using locally weighted scatterplot smoothing with a bandwidth of 2/3. The blue line fitted the HR line at the ends of the breakpoints. ^1^ Tea intake greater than 10 cups treated as 10.

**Fig. S2** Hazard ratios of all-cause neurodegenerative disease and neurodegenerative disease subtypes associated with different levels of coffee intake (vs. no coffee consumption) for participants usually consuming decaffeinated, instant, or ground coffee.

Multivariable model is adjusted for age, sex, smoking statue, drinking statue, sleep duration, education, BMI, stroke history, depression history, overall health status, income, physical activity, CYP1A2. And in assessing the relationship between Alzheimer's disease and coffee intake , the APOE4 gene was additionally adjusted. Tea consumption was also included as a covariate when analyzing the association of coffee consumption with neurodegenerative diseases.

**Fig. S3** Association between coffee and neurodegenerative diseases among CYP1A2 gene subtypes.

Multivariable model is adjusted for age, sex, smoking statue, drinking statue, sleep duration, education, BMI, stroke history, depression history, overall health status, income, physical activity, CYP1A2. And in assessing the relationship between Alzheimer's disease and coffee intake, the APOE4 gene was additionally adjusted. The coffee and tea intake will be mutually adjusted.

**Fig. S4** Association of coffee and tea intake with the risk of incident neurodegenerative disease (Removal of patients with less than 2 years of disease duration)

Coffee and tea intake with the risk of incident a) all-cause neurodegenerative disease, b) primary neurodegenerative disease, c) vascular neurodegenerative disease, d) other neurodegenerative disease, e) Parkinson’s disease, f) Alzheimer's disease, g) vascular dementia, h) amyotrophic lateral sclerosis. Multivariable model is adjusted for age, sex, smoking statue, drinking statue, sleep duration, education, BMI, stroke history, depression history, overall health status, income, physical activity, CYP1A2. And in assessing the relationship between Alzheimer's disease and coffee and tea intake, the APOE4 gene was additionally adjusted. The coffee and tea intake will be mutually adjusted.

**Fig. S5** The nonlinear association of coffee consumption with the risk of incident neurodegenerative disease

Restricted cubic spline for testing the hypothesis of nonlinear correlation between a) all-cause neurodegenerative disease, b) primary neurodegenerative disease, c) vascular neurodegenerative disease, d) other neurodegenerative disease, e) Parkinson's disease, f) Alzheimer's disease, g) vascular dementia, h) amyotrophic lateral sclerosis, and coffee intake. Spline curves represent hazard ratios (HRs) adjusted for age, sex, smoking statue, drinking statue, sleep duration, education, BMI, stroke history, depression history, overall health status, income, physical activity, CYP1A2 and tea intake. And in assessing the nonlinear relationship between Alzheimer's disease and coffee consumption, the APOE4 gene was additionally adjusted. The solid lines are fitted based on Cox-proportional hazard models. The shaded areas show 95% confidential intervals (CIs). And the number of knots is 4.

**Fig. S6** The nonlinear association of tea consumption with the risk of incident neurodegenerative disease

Restricted cubic spline for testing the hypothesis of nonlinear correlation between a) all-cause neurodegenerative disease, b) primary neurodegenerative disease, c) vascular neurodegenerative disease, d) other neurodegenerative disease, e) Parkinson’s disease, f) Alzheimer's disease, g) vascular dementia, h) amyotrophic lateral sclerosis, and tea intake. Spline curves represent hazard ratios (HRs) adjusted for age, sex, smoking statue, drinking statue, sleep duration, education, BMI, stroke history, depression history, overall health status, income, physical activity, CYP1A2 and coffee intake. And in assessing the nonlinear relationship between Alzheimer's disease and tea consumption, the APOE4 gene was additionally adjusted. The solid lines are fitted based on Cox-proportional hazard models. The shaded areas show 95% confidential intervals (CIs). And the number of knots is 4.

**Fig. S7** Association of coffee and tea intake with the risk of incident neurodegenerative disease after additionally adjusting for the number of cigarettes smoked per day

Coffee and tea intake with the risk of incident a) all-cause neurodegenerative disease, b) primary neurodegenerative disease, c) vascular neurodegenerative disease, d) other neurodegenerative disease, e) Parkinson’s disease, f) Alzheimer's disease, g) vascular dementia, h) amyotrophic lateral sclerosis. Multivariable model is adjusted for age, sex, smoking statue, drinking statue, sleep duration, education, BMI, stroke history, depression history, overall health status, income, physical activity, CYP1A2 and the number of cigarettes smoked per day. And in assessing the relationship between Alzheimer's disease and coffee or tea intake, the APOE4 gene is additionally adjusted. The coffee and tea intake will be mutually adjusted.

**Fig. S8** Association of coffee and tea intake with the risk of incident neurodegenerative disease after additionally adding the healthy diet score as a new covariate.

Coffee and tea intake with the risk of incident a) all-cause neurodegenerative disease, b) primary neurodegenerative disease, c) vascular neurodegenerative disease, d) other neurodegenerative disease, e) Parkinson’s disease, f) Alzheimer's disease, g) vascular dementia, h) amyotrophic lateral sclerosis. Multivariable model is adjusted for age, sex, smoking statue, drinking statue, sleep duration, education, BMI, stroke history, depression history, overall health status, income, physical activity, CYP1A2 and healthy diet score. And in assessing the relationship between Alzheimer's disease and coffee or tea intake, the APOE4 gene is additionally adjusted. The coffee and tea intake will be mutually adjusted. ^1^ When analyzing the combined effect of coffee and tea intake on amyotrophic lateral sclerosis, participants who consumed >5 cups of coffee per day and 0-3 cups of tea per day were combined with participants who consumed >5 cups of coffee per day and >3 cups of tea per day in the same group due to the low number of cases in both groups.

**Fig. S9** The nonlinear association of coffee or tea consumption with the phenotypic age acceleration

Restricted cubic spline for testing the hypothesis of nonlinear correlation between a) coffee consumption b) tea consumption and phenotypic age acceleration. Spline curves represent beta adjusted for age, sex, smoking statue, drinking statue, sleep duration, education, BMI, stroke history, depression history, overall health status, income, physical activity, CYP1A2. And coffee and tea intake will be mutual adjusted. The solid lines are fitted based on the beta value derived from the ordinary least squares method. The shaded areas show 95% confidential intervals (CIs). And the number of knots is 5.

**Fig. S10** Association of coffee and tea intake with the risk of incident neurodegenerative disease after retaining participants with missing genetic data.

Coffee and tea intake with the risk of incident a) all-cause neurodegenerative disease, b) primary neurodegenerative disease, c) vascular neurodegenerative disease, d) other neurodegenerative disease, e) Parkinson’s disease, f) Alzheimer's disease, g) vascular dementia, h) amyotrophic lateral sclerosis. Multivariable model is adjusted for age, sex, smoking statue, drinking statue, sleep duration, education, BMI, stroke history, depression history, overall health status, income and physical activity. And the coffee and tea intake will be mutually adjusted.

**Fig. S11** Association of coffee and tea intake with the risk of incident neurodegenerative disease after additional adjustment for medical history of coronary heart disease, cancer, and diabetes.

Coffee and tea intake with the risk of incident a) all-cause neurodegenerative disease, b) primary neurodegenerative disease, c) vascular neurodegenerative disease, d) other neurodegenerative disease, e) Parkinson’s disease, f) Alzheimer's disease, g) vascular dementia, h) amyotrophic lateral sclerosis. Multivariable model is adjusted for age, sex, smoking statue, drinking statue, sleep duration, education, BMI, overall health status, income, physical activity, CYP1A2 and the history of stroke, depression, coronary heart disease, cancer, and diabetes. And in assessing the relationship between Alzheimer's disease and coffee or tea intake, the APOE4 gene is additionally adjusted. The coffee and tea intake will be mutually adjusted.

**Fig. S12** Association of coffee and tea intake with the risk of incident neurodegenerative disease after adjusting for the addition of sugar and milk to coffee and tea.

Coffee and tea intake with the risk of incident a) all-cause neurodegenerative disease, b) primary neurodegenerative disease, c) vascular neurodegenerative disease, d) other neurodegenerative disease, e) Parkinson’s disease, f) Alzheimer's disease, g) vascular dementia, h) amyotrophic lateral sclerosis. Multivariable model is adjusted for age, sex, smoking statue, drinking statue, sleep duration, education, BMI, overall health status, stroke history, depression history, income, physical activity, CYP1A2, sugar addition and milk addition. And in assessing the relationship between Alzheimer's disease and coffee or tea intake, the APOE4 gene is additionally adjusted. The coffee and tea intake will be mutually adjusted.

**Table S1** Neurodegenerative diseases defined according to ICD-9 and ICD-10

|  | ICD-9 | ICD-10 |
| --- | --- | --- |
| all-cause neurodegenerative disease | 290.0, 290.1, 331.0, 331.1, 331.2, 331.9, 332.0, 333.0, 335.2,290.4,290.8,290.9,290.0, 290.1, 331.0 and 332.0 | F00, F02.0, F02.3, G12.2, G20, G23.1, G23.2, G23.8, G23.9, G25.9, G30, G31.0, G31.1, G31.8, G31.9, F01, G21.4, F03, F05.1, G30 and G20 |
| primary neurodegenerative diseases | 290.0, 290.1, 331.0, 331.1, 331.2, 331.9, 332.0, 333.0, and 335.2 | F00, F02.0, F02.3, G12.2, G20, G23.1, G23.2, G23.8, G23.9, G25.9, G30, G31.0, G31.1, G31.8, and G31.9 |
| vascular neurodegenerative diseases | 290.4 | F01, and G21.4 |
| other neurodegenerative diseases | 290.8 and 290.9 | F03, and F05.1 |
| Alzheimer's disease | 290.0, 290.1 and 331.0 | F00, and G30 |
| Parkinson's disease | 332.0 | G20 |
| vascular dementia | 290.4 | F01 |
| amyotrophic lateral sclerosis | 335.20 | G12.2 |

**Table S2** Association of coffee and tea intake with the risk of incident neurodegenerative disease without mutual adjustment of coffee and tea intake.

Multivariable model is adjusted for age, sex, smoking statue, drinking statue, sleep duration, education, BMI, overall health status, income, stroke history, depression history, physical activity and CYP1A2. And in assessing the relationship between Alzheimer's disease and coffee or tea intake, the APOE4 gene is additionally adjusted.

| Disease | | Case | HR (95%CI) | P-value |
| --- | --- | --- | --- | --- |
| All-cause neurodegenerative diseases | |  |  |  |
| Coffee | Tea |  |  |  |
| 0 |  | 1244 | Ref |  |
| 0.5-5 |  | 4829 | 0.99 (0.93-1.05) | 0.681 |
| >5 |  | 410 | 1.20 (1.07-1.34) | 0.002 |
|  | 0 | 948 | Ref |  |
|  | 0.5-3 | 2562 | 0.85 (0.79-0.92) | <0.001 |
|  | >3 | 2973 | 0.82 (0.76-0.88) | <0.001 |
| Primary neurodegenerative diseases | |  |  |  |
| Coffee | Tea |  |  |  |
| 0 |  | 915 | Ref |  |
| 0.5-5 |  | 3739 | 1.02 (0.95-1.10) | 0.592 |
| >5 |  | 306 | 1.23 (1.08-1.40) | 0.002 |
|  | 0 | 701 | Ref |  |
|  | 0.5-3 | 1981 | 0.87 (0.80-0.95) | 0.002 |
|  | >3 | 2278 | 0.84 (0.77-0.91) | <0.001 |
| Vascular neurodegenerative diseases | |  |  |  |
| Coffee | Tea |  |  |  |
| 0 |  | 218 | Ref |  |
| 0.5-5 |  | 737 | 0.93 (0.80-1.09) | 0.364 |
| >5 |  | 71 | 1.16 (0.88-1.52) | 0.293 |
|  | 0 | 170 | Ref |  |
|  | 0.5-3 | 376 | 0.75 (0.62-0.90) | 0.002 |
|  | >3 | 480 | 0.76 (0.63-0.90) | 0.002 |
| Other neurodegenerative diseases | |  |  |  |
| Coffee | Tea |  |  |  |
| 0 |  | 514 | Ref |  |
| 0.5-5 |  | 1884 | 0.97 (0.88-1.07) | 0.525 |
| >5 |  | 158 | 1.15 (0.96-1.38) | 0.126 |
|  | 0 | 377 | Ref |  |
|  | 0.5-3 | 988 | 0.84 (0.75-0.95) | 0.005 |
|  | >3 | 1191 | 0.82 (0.73-0.92) | 0.001 |
| Parkinson’s diseases | |  |  |  |
| Coffee | Tea |  |  |  |
| 0 |  | 340 | Ref |  |
| 0.5-5 |  | 1443 | 1.02 (0.91-1.15) | 0.705 |
| >5 |  | 113 | 1.22 (0.98-1.51) | 0.072 |
|  | 0 | 263 | Ref |  |
|  | 0.5-3 | 781 | 0.89 (0.77-1.02) | 0.105 |
|  | >3 | 852 | 0.83 (0.73-0.96) | 0.011 |
| Alzheimer's diseases | |  |  |  |
| Coffee | Tea |  |  |  |
| 0 |  | 360 | Ref |  |
| 0.5-5 |  | 1481 | 1.03 (0.91-1.16) | 0.642 |
| >5 |  | 123 | 1.28 (1.04-1.58) | 0.019 |
|  | 0 | 269 | Ref |  |
|  | 0.5-3 | 778 | 0.90 (0.79-1.04) | 0.161 |
|  | >3 | 917 | 0.87 (0.76-1.00) | 0.046 |
| Vascular dementia | |  |  |  |
| Coffee | Tea |  |  |  |
| 0 |  | 211 | Ref |  |
| 0.5-5 |  | 710 | 0.93 (0.79-1.08) | 0.346 |
| >5 |  | 68 | 1.15 (0.87-1.51) | 0.33 |
|  | 0 | 164 | Ref |  |
|  | 0.5-3 | 359 | 0.74 (0.61-0.89) | 0.001 |
|  | >3 | 466 | 0.76 (0.64-0.91) | 0.003 |
| Amyotrophic lateral sclerosis | |  |  |  |
| Coffee | Tea |  |  |  |
| 0 |  | 50 | Ref |  |
| 0.5-5 |  | 191 | 0.90 (0.65-1.23) | 0.508 |
| >5 |  | 13 | 0.96 (0.52-1.77) | 0.885 |
|  | 0 | 20 | Ref |  |
|  | 0.5-3 | 107 | 1.58 (0.98-2.55) | 0.062 |
|  | >3 | 127 | 1.67 (1.04-2.67) | 0.035 |

**Table S3**. Subgroup analysis of the association between coffee or tea intake and the risk of incident neurodegenerative disease

Subgroup analysis by grouping participants using a) hypertension and b) diabetes. Multivariable model is adjusted for age, sex, smoking statue, drinking statue, sleep duration, education, BMI, overall health status, income, stroke history, depression history, physical activity and CYP1A2. And in assessing the relationship between Alzheimer's disease and coffee and tea intake more adjusted APOE4 gene. coffee and tea intake will be mutually adjusted.

a)

| **Coffee** | HR (95%CI), P-value | P for interaction | **Tea** | HR (95%CI), P-value | P for interaction |
| --- | --- | --- | --- | --- | --- |
| **All-cause neurodegenerative diseases** |  | 0.020 | **All-cause neurodegenerative diseases** |  | 0.056 |
| Hypertension=No |  |  | Hypertension=No |  |  |
| 0 | Ref |  | 0 | Ref |  |
| 0.5-5 | 0.94 (0.87-1.03),0.173 |  | 0.5-3 | 0.90 (0.81-0.99),0.032 |  |
| >5 | 1.01 (0.86-1.17),0.94 |  | >3 | 0.88 (0.79-0.97),0.008 |  |
| Hypertension=Yes |  |  | Hypertension=Yes |  |  |
| 0 | Ref |  | 0 | Ref |  |
| 0.5-5 | 1.01 (0.91-1.12),0.893 |  | 0.5-3 | 0.84 (0.74-0.94),0.004 |  |
| >5 | 1.31 (1.09-1.57),0.004 |  | >3 | 0.77 (0.68-0.87),<0.001 |  |
| **Primary neurodegenerative diseases** |  | 0.095 | **Primary neurodegenerative diseases** |  | 0.157 |
| Hypertension=No |  |  | Hypertension=No |  |  |
| 0 | Ref |  | 0 | Ref |  |
| 0.5-5 | 0.97 (0.88-1.06),0.487 |  | 0.5-3 | 0.94 (0.84-1.05),0.258 |  |
| >5 | 1.06 (0.90-1.26),0.477 |  | >3 | 0.91 (0.81-1.01),0.083 |  |
| Hypertension=Yes |  |  | Hypertension=Yes |  |  |
| 0 | Ref |  | 0 | Ref |  |
| 0.5-5 | 1.07 (0.94-1.21),0.299 |  | 0.5-3 | 0.83 (0.72-0.96),0.01 |  |
| >5 | 1.34 (1.07-1.67),0.01 |  | >3 | 0.79 (0.68-0.91),0.001 |  |
| **Vascular neurodegenerative diseases** |  | 0.375 | **Vascular neurodegenerative diseases** |  | 0.312 |
| Hypertension=No |  |  | Hypertension=No |  |  |
| 0 | Ref |  | 0 | Ref |  |
| 0.5-5 | 0.88 (0.70-1.09),0.239 |  | 0.5-3 | 0.83 (0.63-1.08),0.161 |  |
| >5 | 0.90 (0.60-1.36),0.623 |  | >3 | 0.85 (0.66-1.11),0.228 |  |
| Hypertension=Yes |  |  | Hypertension=Yes |  |  |
| 0 | Ref |  | 0 | Ref |  |
| 0.5-5 | 0.96 (0.76-1.20),0.708 |  | 0.5-3 | 0.70 (0.54-0.91),0.008 |  |
| >5 | 1.21 (0.82-1.80),0.343 |  | >3 | 0.68 (0.52-0.88),0.004 |  |
| **Other neurodegenerative diseases** |  | 0.209 | **Other neurodegenerative diseases** |  | 0.589 |
| Hypertension=No |  |  | Hypertension=No |  |  |
| 0 | Ref |  | 0 | Ref |  |
| 0.5-5 | 0.95 (0.83-1.08),0.409 |  | 0.5-3 | 0.85 (0.73-1.00),0.05 |  |
| >5 | 0.96 (0.75-1.22),0.723 |  | >3 | 0.84 (0.72-0.98),0.031 |  |
| Hypertension=Yes |  |  | Hypertension=Yes |  |  |
| 0 | Ref |  | 0 | Ref |  |
| 0.5-5 | 0.96 (0.82-1.13),0.664 |  | 0.5-3 | 0.86 (0.71-1.04),0.129 |  |
| >5 | 1.26 (0.94-1.68),0.116 |  | >3 | 0.80 (0.66-0.98),0.028 |  |
| **Parkinson’s diseases** |  | 0.487 | **Parkinson’s diseases** |  | 0.227 |
| Hypertension=No |  |  | Hypertension=No |  |  |
| 0 | Ref |  | 0 | Ref |  |
| 0.5-5 | 1.00 (0.86-1.16),0.962 |  | 0.5-3 | 0.96 (0.80-1.16),0.691 |  |
| >5 | 1.07 (0.80-1.41),0.656 |  | >3 | 0.93 (0.77-1.11),0.412 |  |
| Hypertension=Yes |  |  | Hypertension=Yes |  |  |
| 0 | Ref |  | 0 | Ref |  |
| 0.5-5 | 1.01 (0.82-1.24),0.912 |  | 0.5-3 | 0.82 (0.65-1.03),0.092 |  |
| >5 | 1.29 (0.89-1.85),0.174 |  | >3 | 0.73 (0.58-0.92),0.009 |  |
| **Alzheimer's diseases** |  | 0.689 | **Alzheimer's diseases** |  | 0.654 |
| Hypertension=No |  |  | Hypertension=No |  |  |
| 0 | Ref |  | 0 | Ref |  |
| 0.5-5 | 0.96 (0.83-1.12),0.617 |  | 0.5-3 | 0.95 (0.80-1.14),0.594 |  |
| >5 | 1.17 (0.90-1.53),0.235 |  | >3 | 0.89 (0.75-1.06),0.192 |  |
| Hypertension=Yes |  |  | Hypertension=Yes |  |  |
| 0 | Ref |  | 0 | Ref |  |
| 0.5-5 | 1.13 (0.92-1.39),0.236 |  | 0.5-3 | 0.89 (0.70-1.14),0.364 |  |
| >5 | 1.34 (0.93-1.95),0.119 |  | >3 | 0.93 (0.73-1.18),0.532 |  |
| **Vascular dementia** |  | 0.349 | **Vascular dementia** |  | 0.298 |
| Hypertension=No |  |  | Hypertension=No |  |  |
| 0 | Ref |  | 0 | Ref |  |
| 0.5-5 | 0.89 (0.72-1.12),0.327 |  | 0.5-3 | 0.69 (0.53-0.90),0.006 |  |
| >5 | 0.90 (0.59-1.37),0.615 |  | >3 | 0.68 (0.52-0.89),0.005 |  |
| Hypertension=Yes |  |  | Hypertension=Yes |  |  |
| 0 | Ref |  | 0 | Ref |  |
| 0.5-5 | 0.94 (0.74-1.18),0.575 |  | 0.5-3 | 0.82 (0.63-1.08),0.155 |  |
| >5 | 1.20 (0.81-1.79),0.368 |  | >3 | 0.86 (0.66-1.12),0.26 |  |
| **Amyotrophic lateral sclerosis** |  | 0.711 | **Amyotrophic lateral sclerosis** |  | 0.510 |
| Hypertension=No |  |  | Hypertension=No |  |  |
| 0 | Ref |  | 0 | Ref |  |
| 0.5-5 | 0.94 (0.63-1.42),0.784 |  | 0.5-3 | 1.66 (0.93-2.98),0.088 |  |
| >5 | 1.24 (0.59-2.61),0.567 |  | >3 | 1.57 (0.87-2.82),0.134 |  |
| Hypertension=Yes |  |  | Hypertension=Yes |  |  |
| 0 | Ref |  | 0 | Ref |  |
| 0.5-5 | 0.88 (0.52-1.50),0.646 |  | 0.5-3 | 1.54 (0.63-3.72),0.341 |  |
| >5 | 0.84 (0.24-2.93),0.783 |  | >3 | 2.00 (0.84-4.77),0.119 |  |

b)

| **Coffee** | HR (95%CI), P-value | P for interaction | **Tea** | HR (95%CI), P-value | P for interaction |
| --- | --- | --- | --- | --- | --- |
| **All-cause neurodegenerative diseases** |  | 0.252 | **All-cause neurodegenerative diseases** |  | 0.150 |
| Diabetes=No |  |  | Diabetes=No |  |  |
| 0 | Ref |  | 0 | Ref |  |
| 0.5-5 | 0.96 (0.90-1.03),0.303 |  | 0.5-3 | 0.89 (0.82-0.96),0.005 |  |
| >5 | 1.08 (0.95-1.23),0.236 |  | >3 | 0.86 (0.79-0.93), <0.001 |  |
| Diabetes=Yes |  |  | Diabetes=Yes |  |  |
| 0 | Ref |  | 0 | Ref |  |
| 0.5-5 | 1.00 (0.83-1.21),0.959 |  | 0.5-3 | 0.83 (0.68-1.02),0.073 |  |
| >5 | 1.33 (0.98-1.81),0.064 |  | >3 | 0.74 (0.61-0.91),0.005 |  |
| **Primary neurodegenerative diseases** |  | 0.161 | **Primary neurodegenerative diseases** |  |  |
| Diabetes=No |  |  | Diabetes=No |  | 0.451 |
| 0 | Ref |  | 0 | Ref |  |
| 0.5-5 | 1.00 (0.93-1.09),0.916 |  | 0.5-3 | 0.91 (0.82-1.00),0.043 |  |
| >5 | 1.11 (0.96-1.28),0.173 |  | >3 | 0.88 (0.80-0.97),0.008 |  |
| Diabetes=Yes |  |  | Diabetes=Yes |  |  |
| 0 | Ref |  | 0 | Ref |  |
| 0.5-5 | 1.03 (0.82-1.29),0.829 |  | 0.5-3 | 0.84 (0.66-1.08),0.168 |  |
| >5 | 1.53 (1.06-2.19),0.022 |  | >3 | 0.80 (0.62-1.02),0.074 |  |
| **Vascular neurodegenerative diseases** |  | 0.391 | **Vascular neurodegenerative diseases** |  | 0.473 |
| Diabetes=No |  |  | Diabetes=No |  |  |
| 0 | Ref |  | 0 | Ref |  |
| 0.5-5 | 0.89 (0.75-1.06),0.197 |  | 0.5-3 | 0.83 (0.67-1.03),0.095 |  |
| >5 | 1.13 (0.82-1.54),0.456 |  | >3 | 0.83 (0.67-1.03),0.091 |  |
| Diabetes=Yes |  |  | Diabetes=Yes |  |  |
| 0 | Ref |  | 0 | Ref |  |
| 0.5-5 | 0.98 (0.69-1.39),0.908 |  | 0.5-3 | 0.61 (0.42-0.90),0.012 |  |
| >5 | 0.76 (0.40-1.45),0.399 |  | >3 | 0.66 (0.45-0.96),0.029 |  |
| **Other neurodegenerative diseases** |  | 0.491 | **Other neurodegenerative diseases** |  | 0.370 |
| Diabetes=No |  |  | Diabetes=No |  |  |
| 0 | Ref |  | 0 | Ref |  |
| 0.5-5 | 0.93 (0.84-1.04),0.22 |  | 0.5-3 | 0.85 (0.74-0.97),0.017 |  |
| >5 | 1.06 (0.87-1.30),0.552 |  | >3 | 0.84 (0.74-0.96),0.01 |  |
| Diabetes=Yes |  |  | Diabetes=Yes |  |  |
| 0 | Ref |  | 0 | Ref |  |
| 0.5-5 | 1.07 (0.81-1.42),0.618 |  | 0.5-3 | 0.96 (0.71-1.30),0.796 |  |
| >5 | 1.11 (0.68-1.82),0.675 |  | >3 | 0.83 (0.61-1.14),0.248 |  |
| **Parkinson’s diseases** |  | 0.317 | **Parkinson’s diseases** |  | 0.364 |
| Diabetes=No |  |  | Diabetes=No |  |  |
| 0 | Ref |  | 0 | Ref |  |
| 0.5-5 | 0.98 (0.86-1.11),0.768 |  | 0.5-3 | 0.93 (0.79-1.08),0.32 |  |
| >5 | 1.10 (0.87-1.39),0.434 |  | >3 | 0.88 (0.76-1.03),0.105 |  |
| Diabetes=Yes |  |  | Diabetes=Yes |  |  |
| 0 | Ref |  | 0 | Ref |  |
| 0.5-5 | 1.29 (0.84-1.99),0.245 |  | 0.5-3 | 0.78 (0.52-1.18),0.248 |  |
| >5 | 1.56 (0.80-3.04),0.19 |  | >3 | 0.66 (0.43-1.02),0.061 |  |
| **Alzheimer's diseases** |  | 0.497 | **Alzheimer's diseases** |  | 0.904 |
| Diabetes=No |  |  | Diabetes=No |  |  |
| 0 | Ref |  | 0 | Ref |  |
| 0.5-5 | 1.03 (0.91-1.17),0.609 |  | 0.5-3 | 0.94 (0.80-1.09),0.41 |  |
| >5 | 1.21 (0.96-1.53),0.108 |  | >3 | 0.91 (0.78-1.06),0.228 |  |
| Diabetes=Yes |  |  | Diabetes=Yes |  |  |
| 0 | Ref |  | 0 | Ref |  |
| 0.5-5 | 0.90 (0.64-1.27),0.563 |  | 0.5-3 | 0.92 (0.62-1.36),0.688 |  |
| >5 | 1.32 (0.75-2.33),0.338 |  | >3 | 0.94 (0.64-1.39),0.759 |  |
| **Vascular dementia** |  | 0.430 | **Vascular dementia** |  | 0.545 |
| Diabetes=No |  |  | Diabetes=No |  |  |
| 0 | Ref |  | 0 | Ref |  |
| 0.5-5 | 0.89 (0.74-1.07),0.205 |  | 0.5-3 | 0.82 (0.66-1.02),0.076 |  |
| >5 | 1.12 (0.81-1.55),0.494 |  | >3 | 0.83 (0.67-1.04),0.104 |  |
| Diabetes=Yes |  |  | Diabetes=Yes |  |  |
| 0 | Ref |  | 0 | Ref |  |
| 0.5-5 | 0.97 (0.69-1.38),0.883 |  | 0.5-3 | 0.62 (0.42-0.91),0.015 |  |
| >5 | 0.77 (0.40-1.47),0.432 |  | >3 | 0.67 (0.46-0.98),0.04 |  |
| **Amyotrophic lateral sclerosis** |  | 0.058 | **Amyotrophic lateral sclerosis** |  | 0.183 |
| Diabetes=No |  |  | Diabetes=No |  |  |
| 0 | Ref |  | 0 | Ref |  |
| 0.5-5 | 1.06 (0.75-1.50),0.749 |  | 0.5-3 | 1.79 (1.05-3.07),0.034 |  |
| >5 | 1.22 (0.61-2.42),0.57 |  | >3 | 1.99 (1.17-3.40),0.012 |  |
| Diabetes=Yes |  |  | Diabetes=Yes |  |  |
| 0 | Ref |  | 0 | Ref |  |
| 0.5-5 | 0.22 (0.08-0.61),0.004 |  | 0.5-3 | 0.92 (0.27-3.15),0.893 |  |
| >5 | 0.62 (0.12-3.24),0.568 |  | >3 | 0.42 (0.11-1.61),0.204 |  |
